# Fellowships, Grants, & Awards

**Published:** 2006-11

## Parenting Capacities and Health Outcomes in Youths and Adolescents (R21)

This funding opportunity announcement (FOA) solicits research aimed at increasing parenting capacities while simultaneously focusing on the reduction, elimination, or prevention of one or more high-risk health behaviors or poor health habits in youth and adolescent children. Investigators responding to this announcement are required to target two or more ineffective parenting practices or behaviors (e.g., lack of appropriate parental monitoring, supervision, and communication, high family conflict and disorganization, parental stress and depression, lack of parent–child bonding and negative discipline methods), and two or more youth/adolescent high-risk behaviors [e.g., unhealthy dietary behaviors, inadequate physical activity, tobacco use, alcohol and other drug use, sexual behaviors, and unintentional (accidents) and intentional behaviors (firearm related injuries]. These behaviors are frequently established during childhood and adolescent years and continue on to the adult years. These behaviors are potentially amenable to a variety of health promotion and prevention efforts. As part of the national efforts to eliminate health disparities, proposals specifically targeting racial/ethnic minority populations are strongly encouraged. Strategic Plans on Reducing Health Disparities are located at http://www.nih.gov/ninr, http://www.nhlbi.nih.gov/resources/docs/plandisp.htm, http://www.niaaa.nih.gov/, http://www.nichd.nih.gov/, and http://www.nida.nih.gov/.

Research targeting other diverse groups of parents and their youths/adolescents is also encouraged (e.g., sexual orientation, religious background, single-parent families). For purposes of this announcement, children 10 to 18 years of age can be targeted.

It is well documented that the probability of children and adolescents acquiring nonoptimal health behaviors and developmental problems increases substantially in the presence of ineffective parenting practices. Specifically, there is empirical support for the notion that many early precursors of serious middle-childhood and adolescent problems can be significantly reduced or thwarted through effective early interventions aimed at improving parenting practices and family dynamics. Although it would be gross oversimplification to equate child and adolescent developmental, behavioral, and health outcomes to products of child-rearing practices, parental and care-giver behaviors and practices do have a large impact and exert perhaps the most significant and pervasive influence over the health risk behaviors and health habits of their adolescent children.

Undoubtedly, genes, peers, the mass media, school, neighborhood, and other societal and cultural influences do play a role in shaping child and adolescent developmental, behavioral, and health outcomes. Nevertheless, the critical roles of parenting and the family dynamics cannot be minimized. There is convincing evidence that parenting behaviors profoundly impact the development of positive behaviors and outcomes in youths and adolescents. Parental behaviors implicated include appropriate parental monitoring, supervision, and communication, low family conflict and disorganization, low parental stress and depression, healthy parent–child bonding and positive discipline methods (evidenced by interactional behaviors associated with warmth, patterns of punishment and reward, verbal techniques, appropriate direction and control). For example, although evidence suggests that peer influence is pivotal in the initiation of negative behaviors in youths and adolescents, parental disapproval has been shown to have a major suppressive effect for alcohol and drug use. Other recent evidence also suggests that a positive family environment, including positive parent–child relationships, consistent supervision, and communication of prosocial and healthy values and expectations, act as major deterrents for youths and adolescents to engage in negative and unhealthy behaviors and are associated with better school performance and psychosocial development.

A 2003 report from the National Academy of Sciences revealed that between 1970 and 2000 the maternal labor force rose from 38 to 68%. Moreover, this trend reflects a broad range of demographics and circumstances, including first-time and, never-married mothers, mothers of all income brackets, education levels, race, ethnicity, or place of residence. With parents spending substantial amounts of their daily lives at the workplace, and with fewer supports from a second parent or extended family members, coupled with high adolescent and youth involvement in negative and unhealthy behaviors, parents need to know how to parent effectively. Parenting effectively includes, in addition to the aforementioned factors, an understanding of the concept of healthy adolescence and normative development, how to assess the status of their adolescent child in terms of his or her health trajectory, how to facilitate healthy development and foster the adoption of positive health behaviors, and how to access resources and support for themselves and their adolescent when there is a problem. Adolescent health behaviors include, but are not limited to, nutrition/dietary practices, physical activity, weight control, drug, alcohol, and tobacco use, injuries and violence, and sexual activity.

In 2004, > 40 million U.S. residents were classified as adolescents ranging in age from 10–19 years. This represents 14% of the U.S. population. Among these, approximately 2/3 of the adolescent population were non-Hispanic and 1/3 of other racial ethnic identity. Projections indicate that by year 2050, the nation's racial ethnic minority groups (black, Hispanic, American Indian, Asian) will constitute approximately 56% of the adolescent population. While the health status of adolescents differs according to age, sex, race, and ethnic origin, there is ample documentation suggesting that adolescents, regardless of background, engage in high-risk behavior.

Today, tobacco use constitutes the single leading cause of preventable death in the United States. Epidemiologists estimate that tobacco-related illnesses will be responsible for > 5 million premature deaths among persons ≤ 17 years of age who began to use tobacco products in 1995. Data from the Youth Risk Behavior Surveillance System (YRBS) noted that 80% of individuals who use tobacco began before age 18. Further, data from the 2004 Monitoring the Future (MTF) Survey of 50,000 students show that 9% of 8th graders reported smoking in the prior 30 days, along with 16% of 10th graders and 25% of 12th graders. Some important subgroup differences have been observed, particularly for smoking. Students who plan to complete a 4-year college education are much less likely to smoke than those who do not have such plans. Youth living in rural areas and small town areas are considerably more likely to smoke than those living in metropolitan areas. Students with more educated parents are less likely to smoke, particularly at young ages.

Poor dietary habits or improper eating habits have been linked to the development of coronary heart disease, cancer, diabetes, osteoporosis, hypertension, and obesity. Although childhood obesity has been a longstanding public health problem, recent increases have raised the level to epidemic proportions among U.S. children. A 2005 IOM report, estimated that approximately 9 million children > 6 years of age were overweight. Among children 6–19 years, 31% were at risk for overweight or overweight, and 16% were overweight (BMI > 95th percentile for age growth charts). These findings, based upon measured weight and height, reflect a substantial and alarming increase in childhood obesity over the past two decades. Further, the current increase is most prominent among non-Hispanic black and Mexican-American children, with the increase in these groups at > 10% over the last two decades. For example, for non-Hispanic black and Mexican-American adolescents, overweight prevalence increased from 13.4 to 23.6% and from 13.8 to 23.4%, respectively. Despite recommendations for a healthier diet, recent data show that the usual diet of today's adolescents includes foods high in saturated fat, high in calorie-dense foods, and low in fruit and vegetable consumption.

Similarly, inadequate physical activity contributes to a variety of adverse health conditions and consequences. In contrast, regular physical activity has been associated with increased psychological and mental well-being, improved cardiovascular health, and proper weight maintenance. Physical activity declines during the transition from childhood through adolescence. The National Growth and Health Studies found a 34% decline in physical activity in youth during this transitional period from 10–18 years of age. The decline is more dramatic in girls and in African-American youth. Data from the YRBS found that vigorous physical activity declines about 30% between grades 9 and 12. Thus, the decline in physical activity begins early, about the time of entry into middle school, and continues throughout adolescence.

This decline in physical activity may contribute to the recent increases in obesity among adolescents. Physical inactivity during adolescence may later result in obesity during adulthood. Healthy People 2010 and the Dietary Guidelines for America recommends that adolescents engage in physical activity that promotes cardiovascular fitness most days of the week for a minimum of ≥ 60 min per occasion.

With respect to substance abuse, the use of alcohol and other drugs was among the four risk behaviors of concern among adolescents during the 1990s and continue to be major public health concerns today. Underage drinking particularly, is a major public health problem. Alcohol is the drug of choice for youth, and, on average, adolescents tend to drink more per occasion than adult drinkers, with binge drinking (≥ 4 drinks for women, ≥ 5 for men) especially problematic. In a 2003 IOM and NRC report, it was revealed that nearly half of high school seniors reported using alcohol 30 days prior to being surveyed; this compares to 27% for tobacco and 22% for marijuana. Most adolescents being surveyed reported starting using alcohol or other drugs before entering high school. An additional troubling finding is that the proportion of underage drinkers has remained virtually unchanged from 2002. The use of alcohol and other drugs is associated with a variety of adverse consequences such as violence, injuries in car crashes, and premature death. In addition, national survey data show that early initiation of alcohol use is associated with elevated risk for later alcohol dependency. The IOM and NRC concluded new interventions such as those targeting parents and other adults are warranted. Finally, according to the 2004 MTF survey, several illicit drugs (such as marijuana, ecstasy, and amphetamines) have shown modest declines, while other drugs have been showing signs of increasing use. The main areas of concern that are raised by the 2004 MTF findings are the increasing use of inhalants by 8th graders, and the continued high rates of abuse of prescription painkillers in each grade.

Responsible sexual behavior has been identified as a leading health indicator in Healthy People 2010, with specific behavior objectives aimed at increasing the proportion of adolescents who abstain from sexual intercourse or use condoms if sexually active. Adolescents who engage in early sexual behaviors are at an increased risk of sexually transmitted diseases including HIV infection as well as unintended pregnancy, and, for drinking teens, possibly conceiving a child afflicted with Fetal Alcohol Spectrum Disorder (FASD). Recent estimates (2005) are that over half of all high school students engage in some form of sexual activity. However, national trends have also showed that the prevalence of sexual experience among adolescents decreased 8% while the prevalence of multiple partners decreased 13%.

Currently, motor vehicle and firearm-related injuries are the leading cause of death for adolescents. The high death rates from motor vehicle injuries are partly due to high-risk behaviors among adolescents (e.g., drunken driving, the nonuse of seat belts). Motor vehicle death rates were higher for male adolescents, non-Hispanic white, American Indian, and Alaskan Native adolescents and lower among non-Hispanic black, Hispanic, Asian, and Pacific Islander adolescents. Death rates from firearm injuries increase with age, with highest rates for males ≥ 19 years of age. Similarly, death rates from firearm injuries for females ≥ 19 years of age are 10 times higher when compared with firearm death injuries for females 11–18 years. Nevertheless, firearm-related death rates are strikingly higher for black adolescents when compared to adolescents from other racial/ethnic minority groups. These two leading causes of death are identified as high-priority areas in Healthy People 2010.

In summary, scientific evidence suggests that interventions targeting parents and families can exert powerful influence and are cost-effective in reducing or eliminating health risk behaviors among adolescent children. Continued research is needed to decrease the numbers of adolescents engaging in high-risk behaviors, thus reducing and ameliorating the short- and long-term consequences associated with these behaviors. Further, many of these behaviors are interrelated and thus may be amenable to interventions that address multiple risk behaviors simultaneously. For example, research has shown that youths and adolescents who engage in heavy drinking may also engage in high-risk sexual behaviors. Thus, targeting both ineffective parenting practices and high-risk behaviors in one application may be more successful in improving the overall health profile of youths and adolescents. Research has shown that interventions that target combinations of risk factors may result in more successful and long-term behavioral changes. Similarly, more research is needed that takes into consideration parental characteristics (e.g., age, sex, and socioeconomic status), family dynamics, geographic location, and other special needs that may affect the promotion of adolescent behavior and health outcomes. Psychosocial mediators of health behavior such as parental cultural beliefs and values and parental self-efficacy must also be considered. Applications in response to this announcement should be grounded in theory and should reflect the current literature on parenting and its effects on adolescent risk behaviors. Applications should identify constructs to be measured, review the relevant theoretical literature, and clearly identify research aims and design, along with the strengths and limitations of the proposal. Outcome measures should have sound psychometric properties and should be age- and language-appropriate. The cultural and socioeconomic status of participants should also be considered.

The following are potential areas of research related to this program announcement. These examples are not listed in any priority and are not to be viewed as exhaustive or an exclusive listing of potential areas. Suitable topics for research include, but are not limited to: 1) interventions that incorporate protective factors that aid in improving parenting practices and preventing youth and adolescents from engaging in multiple risky behaviors; 2) interventions that focus on modifying parental practices to improve diet and physical activity and prevent excessive weight gain in normal weight youth and/or reduce rate of weight gain in overweight youth; 3) interventions testing the effects of modifying parental psychosocial factors (e.g., parental self-efficacy) on diet and physical activity behaviors with the goal of controlling childhood obesity; 4) biobehavioral descriptive or intervention studies that elucidate and incorporate the physiological, psychological, socioeconomic, emotional, environmental, cultural, and genetic factors that influence parenting practices and health compromising and or health promoting behaviors among youths and adolescents; 5) innovative intervention studies devoted to enhancing parent self-efficacy, competence, and skill development to support the initiation and or maintenance of youth and adolescent health-promoting behaviors; 6) intervention studies using community-based approaches to facilitate improved parenting practices and health promotion/risk reduction behaviors in youth and adolescents in rural and urban settings. Community interventions using indigenous community infrastructures are relevant, as is the fostering of partnerships between academic researchers and community health care providers. Reaching populations outside of traditional channels (such as families involved with social service agencies, the juvenile justice or foster care systems) may identify parents and children with clusters of risk factors; 7) prevention/intervention strategies that target parents or caregivers of youth and adolescents with HIV/AIDS; 8) prevention/intervention strategies that target families with special needs, such as parents or caregivers of youths and adolescents with chronic illness or developmental disabilities; 9) studies investigating the effects and interrelationships among psychosocial and environmental factors (e.g., single parenting, sex, age, poverty, geographic location), and the adoption of high-risk behaviors or health-promoting behaviors among youth and adolescents; 10) intervention studies that facilitate parent/child communication and bonding in situations where child/parent temperaments conflict leading to difficulties with discipline and subsequent risk behaviors; 11) prevention studies that incorporate motivational strategies for behavior change and skills development for behavioral control for children (and parents); 12) culturally and linguistically appropriate intervention studies that incorporate the stages of cognitive and normative development in families having diverse backgrounds; 13) unique and culturally sensitive interventions to promote healthier dietary intake and adequate activity in minority parents and their youth and adolescents; 14) interventions to reduce the risk of poor outcomes for children in families affected by alcohol use disorders. This can include intervening with parents whose alcohol use is impeding effective caregiving; youth with experimental or established alcohol use patterns who need improved parental attention; parents or youth with neurological damage and/or behavioral and cognitive deficits resulting from prenatal alcohol exposure who are at high risk for future social, psychological, and psychiatric problems; 15) prevention strategies targeting a broad range of parenting factors, focusing on family system changes during late childhood and adolescence; 16) intervention strategies focusing on multiple risk or protective parenting and caregiver practices in vulnerable families (such as single mothers and fathers, grandparent(s) as parents/caregivers, low income, migrant, parents with chronic illnesses such as depression or HIV/AIDS, parents with a history of substance use or abuse, etc.) that contribute to or protect against negative youth and adolescent and health behavior outcomes; 17) testing of prevention strategies, known to be effective with early and middle childhood, adapted for use with older youth and adolescents; 18) worksite-based intervention studies to facilitate improved parenting practices and health promotion/risk reduction behaviors in youths and adolescents in rural and urban settings; 19) biobehavioral or intervention strategies that represent improved or new methods (e.g., adaptation of or novel technology) and or measures that are culturally appropriate; 20) prevention interventions that target parenting practices and training in drug education in combination with youth and adolescent drug abuse prevention strategies, during key transition points, such as the transition to middle school or high school; 21) novel or adapted drug abuse prevention programs that are tailored to racial and ethnic minority groups, and are culturally appropriate and theoretically based.

This FOA will use the NIH Exploratory/Developmental Research Grant (R21) award mechanism. As an applicant, you will be solely responsible for planning, directing, and executing the proposed project.

This FOA uses just-in-time information concepts. It also uses the modular budget formats (see the Modular Applications and Awards section of the NIH Grants Policy Statement. Specifically, if you are submitting an application with direct costs in each year of $250,000 or less (excluding consortium Facilities and Administrative [F&A] costs), use the PHS398 Modular Budget component provided in the SF424 (R&R) Application Package and SF424 (R&R) Application Guide (see specifically Section 5.4, “Modular Budget Component,” of the Application Guide).

Exploratory/developmental grant support is for new projects only; competing renewal (formerly “competing continuation”) applications will not be accepted. Up to two resubmissions (formerly “revisions/amendments") of a previously reviewed exploratory/developmental grant application may be submitted. See NOT-OD-03-041, May 7, 2003.

Applicants must download the SF424 (R&R) application forms and SF424 (R&R) Application Guide for this FOA through Grants.gov/Apply.

Note: Only the forms package directly attached to a specific FOA can be used. You will not be able to use any other SF424 (R&R) forms (e.g., sample forms, forms from another FOA), although some of the Attachment files may be useable for more than one FOA.

For further assistance, contact GrantsInfo: 301-435-0714 (telecommunications for the hearing impaired: TTY 301-451-0088) or by e-mail GrantsInfo@nih.gov.

The application submission dates are available at http://grants.nih.gov/grants/funding/submission-schedule.htm. The complete version of this PA is available at The complete version of this PA is available at http://grants.nih.gov/grants/guide/pa-files/PA-06-530.html.

Contacts: The complete list of agency contacts is available at http://grants.nih.gov/grants/guide/pa-files/PA-06-298.html.

Reference PA-06-530.

Reference PA-06-530.

